# Use of artificial intelligence for classification of fractures around the elbow in adults according to the 2018 AO/OTA classification system

**DOI:** 10.1186/s12891-025-09161-2

**Published:** 2025-09-09

**Authors:** Annelie Pettersson, Michael Axenhus, Teo Stukan, Oscar Ljungberg, Hans Nåsell, Ali Sharif Razavian, Max Gordon

**Affiliations:** Department of Clinical Sciences at Danderyds Hospital, Department of Orthopedic Surgery, Karolinska Institutet, Stockholm, 182 88 Sweden

**Keywords:** Artificial intelligence, Radiography, Deep learning, Radiographic image interpretation, computer-assisted

## Abstract

**Background:**

This study evaluates the accuracy of an Artificial Intelligence (AI) system, specifically a convolutional neural network (CNN), in classifying elbow fractures using the detailed 2018 AO/OTA fracture classification system.

**Methods:**

A retrospective analysis of 5,367 radiograph exams visualizing the elbow from adult patients (2002–2016) was conducted using a deep neural network. Radiographs were manually categorized according to the 2018 AO/OTA system by orthopedic surgeons. A pretrained Efficientnet B4 network with squeeze and excitation layers was fine-tuned. Performance was assessed against a test set of 208 radiographs reviewed independently by four orthopedic surgeons, with disagreements resolved via consensus.

**Results:**

The study evaluated 54 distinct fracture types, each with a minimum of 10 cases, ensuring adequate dataset representation. Overall fracture detection achieved an AUC of 0.88 (95% CI 0.83–0.93). The weighted mean AUC was 0.80 for proximal radius fractures, 0.86 for proximal ulna, and 0.85 for distal humerus. These results underscore the AI system’s ability to accurately detect and classify a broad spectrum of elbow fractures.

**Conclusions:**

AI systems, such as CNNs, can enhance clinicians’ ability to identify and classify elbow fractures, offering a complementary tool to improve diagnostic accuracy and optimize treatment decisions. The findings suggest AI can reduce the risk of undiagnosed fractures, enhancing clinical outcomes and radiologic evaluation.

**Supplementary Information:**

The online version contains supplementary material available at 10.1186/s12891-025-09161-2.

## Introduction

The elbow joint consists of the proximal radioulnar joint (PRU), the humeroulnar joint, and the humeroradial joint [1]. Fractures affecting the elbow constitute around 5% of all fractures in adults [2]. Some fracture patterns may seem inconspicuous but pose a great threat to the patient’s well-being. Delayed treatment due to missed fractures, or misinterpretation of the severity of the injury in plain radiographs, results in a greater loss of motion and higher risk for post-traumatic arthritis than if the injuries are treated in a timely manner [3, 4]. Based on our hypothesis and supported by findings in previous publications, an AI system has the potential to aid clinicians in identifying and describing these fractures more accurately, which can help avoid delayed treatment and potential complications such as loss of motion or post-traumatic arthritis.

Reliable diagnosis and description of fractures are crucial for providing correct treatment directly, hence avoiding complications. Computerized tomography (CT) might improve accuracy in some cases, but is not as readily available as plain radiographs which remain the first-line assessment in acute situations [2]. We believe that computer-aided interpretation of radiographs could be greatly useful in both helping clinicians accurately assess the fracture as well as in retrospectively analyzing large amounts of fracture images to improve treatment regimens. This could prove greatly beneficial given the increasing volume of medical images that clinicians need to interpret and analyze.

Promising results have been shown in recent studies in applying a form of artificial intelligence (AI) called deep neural networks for image interpretation [5]. Several studies have been conducted on deep learning for fracture recognition with highly promising results [6–10], although studies on clinical applications and limitations are still few [11].

There is, to our knowledge, no study applying deep learning to encompass both the detection and classification of all types of elbow fractures in adults; and only a few published on fracture classification [9, 11–13]. In addressing the existing body of literature, it becomes evident that while prior studies have made significant strides in applying AI to orthopedic imaging, a comprehensive analysis encompassing all types of elbow fractures in adults remains notably scarce. Our study aimed to evaluate both the ability of the AI system to detect fractures, by distinguishing between “fracture” and “no fracture”, and to classify them into detailed subcategories as per the 2018 AO/OTA classification system. This dual focus ensures that the model addresses both the initial diagnostic need of identifying fractures and the subsequent classification of their specific types. By combining these capabilities, the study offers a comprehensive assessment of the AI system’s potential utility in clinical workflows for elbow fracture management. The uniqueness of our study lies in its tailored scope, aiming to fill a critical gap in the current literature and provide valuable insights for improving clinical practices in the diagnosis and treatment of elbow fractures. Overall, the benefits of integrating AI systems for both fracture detection and classification include improved diagnostic accuracy, significant time savings, and the potential for optimized treatment regimens, all of which ultimately contribute to better patient outcomes.

## Materials and methods

### Ethical approval

The research was approved by the Swedish Ethical Review Authority (DNR: 2014/453 − 31) (The Swedish Ethical Review Authority). Consent to participate was waived due to the study being performed on anonymized data.

### Study design and setting

This study aims to validate a diagnostic method based on retrospectively collected radiographic examinations. These examinations were analyzed by a deep neural network for both presence and type of fracture. In this study a fracture involving the elbow joint is defined as any fracture of the proximal ulna, proximal radius, or distal humerus, thus affecting the elbow joint.

### Data selection

Between the years 2002 and 2016, all exams visualizing the elbow taken of adult patients, and their corresponding radiology reports were collected from Danderyd University Hospital, Stockholm, Sweden. Both the images and their radiology reports were anonymized. Double review of image diagnostics is always carried out at Danderyd University Hospital, and the second review is always done by a licensed radiologist with more than 8 years of experience in musculoskeletal imaging to ensure diagnostic consistency. Phrases suggested fractures affecting the elbow joint of certain fracture subtypes (based off of AO/OTA classification system, including various subgroups and modifiers) were identified using radiology reports, including both the native language (Svenska) and English. Phrases include, but are not exclusive to, “armbågsfraktur”, “olecranonfraktur”, “caput radiifraktur”, “utgjutning”, “felställning”.

A random subset of image series was then selected, including both images with phrases suggesting the presence and absence of a fracture. Initially, regular expressions were applied to the radiology reports to identify terms indicative of fractures, such as “comminuted,” which helped prioritize rarer and more complex fracture types. Due to variability in the accuracy and specificity of the radiology reports, additional strategies were employed to refine the dataset as the project advanced, including active learning, often targeting both rare and ambiguous cases. These two challenges frequently overlapped, as rare fracture types tended to show lower model performance and less distinct visual features.

Once preliminary models were trained and demonstrated a capacity to classify fractures, we adopted a model-driven selection process referred to as active learning. This approach utilized the model’s predictions to strategically identify cases for inclusion. Specifically, we focused on two categories:

1. High-confidence predictions for rare classes: A large volume of exams was scanned, and cases with a high probability of belonging to underrepresented categories were selected manually to ensure balanced representation.
2. Low-confidence predictions for ambiguous cases: Exams with intermediate probability scores (e.g., around 50%) were prioritized to address instances where the model struggled to differentiate between similar classes, thereby targeting areas of classification weakness.

This iterative method of data selection allowed the network to improve its ability to identify and classify challenging or uncommon fracture types, aligning with the principle of active learning. Although traditional active learning often emphasizes cases near the 50% probability margin, we found that this strategy was only effective in cases where the model already demonstrated a foundational understanding. We did not use a strict threshold, but categories with AUC < 0.6 were seen as lacking foundational understanding. In these cases, ~ 50% predictions were often just noise. For rarer or poorly understood categories, a hybrid approach that combined high-confidence predictions and manual selection proved more efficient.

By employing this multi-step selection process, we ensured that the dataset was both diverse and representative of the fracture subtypes relevant to the study. This strategy also mitigated the risks associated with imbalanced data, which could otherwise bias the model’s performance toward more common fracture types. Radiographic projections were not standardized and forearm and diaphyseal humerus protocols were included as they display the elbow joint. Most radiographs followed standard projections, and standard DICOM grayscale normalization was applied. We did not exclude non-standard views, as in clinical practice projections may vary due to patient pain or staff inexperience. Only the initial exam within 90 days of each patient was included to avoid duplication of cases at different stages preventing an overestimation of the network. Series of images where the quality was considered too poor to distinguish fracture lines were excluded. Images of elbow fractures in children with open physes were also excluded by the reviewer as they are classified differently.

The input (radiographic images), as well as the information of expected output label (classification of fractures) are fed to the network, hence establishing a connection between specific fracture features and corresponding categories with the use of supervised learning [5].

### Method of classification

Before being fed to the network, all exams and corresponding radiology reports were labeled according to AO/OTA-class (v. 2018) using a custom-made platform by two senior orthopedic surgeons working with trauma orthopedics, MG (Minimum 10 years of experience) and HN (Minimum 20 years of experience), along with the contribution of two residents, AP and OL, at the orthopedic surgery department at Danderyd University Hospital. The AO/OTA classification system was chosen since it applies to all segments of the elbow joint [14] and because of its level of detail. All fractures were classified in detail down to the lowest distinguishable subgroup, or qualifier. For a subset of fractures, we included a displaced class, AOOTA modifier number 2. We included custom classes such as “arthritis” (further subdivided into severity), where radiographic findings such as erosions (focal discontinuity of the thin, white, subchondral bone plate), joint-space narrowing, demineralization, malalignment, soft tissue swelling, subluxation, and ankylosis were used to determine the severity of arthritis. The findings were identified as inflammatory if the radiologist’s report indicated this or if there were clear erosions and bone destructions not common for osteoarthritis. Additionally, we incorporated the “implant/no implant” class, further divided into types, including IM-nails, screws, plates, cerclage, and arthroplasties. Old fractures were also present in the data sets and were categorized separately due to difficulties in determining fracture type and to prevent the AI from learning incorrectly from those old fractures. The term “old fracture” was defined as any bone deformity that could stem from a healed previous fracture and display characteristics such as delayed union, malunion, or nonunion of fractures. Additionally, a custom class identified whether effusion could be observed to assess how well the network could recognize these qualities regardless of AO/OTA class. After the data sets were labeled by the aforementioned surgeons and residents, they were then fed into the network. Only the test-set was double-read, and the training data was read by a single researcher.

The data was randomly divided into three sets: test, training, and validation. This division was constructed so that a patient appearing on multiple occasions could be included several times in the same set, but without overlap between the three sets.

### Data sets

The test set consisted of 208 cases, which were classified by MG and HN independently. Any discrepancy or disagreement was dealt with during a joint reevaluation session until a consensus was reached. The test set then worked as the common reference against which the final network was tested. For a class to be included in the test set, images had to visualize the elbow and have at least an anteroposterior and a lateral view of the joint.

Two sets of images were used during training; the training set from which the network learned, and a validation set for fine-tuning network parameters, choosing labeling strategies, and evaluating performance.

For the training set, we initially selected exams at random from the categories identified in the radiologist’s reports for classification. As the learning processes advanced, cases were selected based on network output: (a) cases with a high probability of a class were selected in each category, and then (b) cases where the network was most indecisive were used, i.e. active learning [4]. Due to the high number of available classes the selected category was altered depending on which categories were performing inadequately at that stage. Some of the labeled images were revisited during the labeling if the reviewer had marked them for revisit or if the network had issues with that category.

### Neural network setup

The network used is a modification of an open-source pre-trained Efficientnet B4 network with squeeze and excitation layers [15] where each image was fed through the core network where the images were merged to exam-level by taking the mean of the top 50% features. These values were then fed through a 2-layer fully connected network to provide a per-label prediction. We used a margin-loss where labels that would have not been eligible were ignored, e.g. if the label proximal radius fracture was not present the AO-classes would be missing instead of negative. All images retained their original proportions and we did not stretch images to the full canvas size.

The network was initially trained without any disturbance before data augmentation was implemented during training, including white noise, random deletion of five blocks (20 × 20 pixels each) per image, and stochastic weighted averaging in a sequential fashion [16]. During training, we used additional exams for other anatomical sites that considered similar tasks, i.e. ankle fracture and knee fracture datasets [12, 17].

All available radiographs in each series were presented to the network and each radiograph was automatically cropped to the area of the active image, i.e. black borders were removed, and the image was reduced to a maximum of 256 pixels. Padding was then added to the rectangular image so that the network received a square format of 256 × 256 pixels.

Area under the curve (AUC) was measured as the primary outcome for network performance while sensitivity and specificity were used as secondary outcome measures. The proportions of correctly detected fractures were estimated using AUC – the area under a receiver operating curve (ROC) – a plot of true positive rate against false positive rate and suggests the network’s ability to sort the class according to likelihood, from low to high. An AUC value of 1.0 suggests that the network is always correct and a value ≤ 0.5 that it is no better than random chance. An AUC of < 0.7 is generally considered poor, 0.7–0.8 as acceptable, 0.8–0.9 as good to excellent, and ≥ 0.9 is considered outstanding, however, there is no exact guide for how to interpret AUC values [18–20]. Inter-rater agreement estimates were performed using Cohen’s kappa for key classes.

Since there are many categories, we also presented a frequency-weighted average that included the subclasses of each measure; e.g. B-types will not only include the B-type but also all available groups and subgroups into one measure. The weighting was done according to the number of positive cases since we wanted to avoid small categories, which may have performed well by chance, having a large impact on the frequency-weighted average. AUC was calculated:

$$AUC_{weighted\;=\;}\frac{\sum_{i=1}^{categories}AUC_in_i}{\sum_{i=1}^{categories}\;n_i}$$

We implemented integrated gradients as a method to assess which image features the network analyzed to arrive at its output since this is not otherwise immediately accessible. This information is displayed by integrated gradients as a heat map where the color red illustrates image features that contribute positively to a certain output, i.e. the fracture category, and blue illustrates features that contribute against the same output [15]. Additional measurements such as Youden J and prAUC are provided in the supplement. 

The network was implemented and trained using PyTorch (v. 1.10) and statistical analysis was performed using R (4.2.1) The logical algorithm of the study is described in the supplementary material (Supplemental Fig. 1).

## Results

### Data extraction

**Fig. 1:**
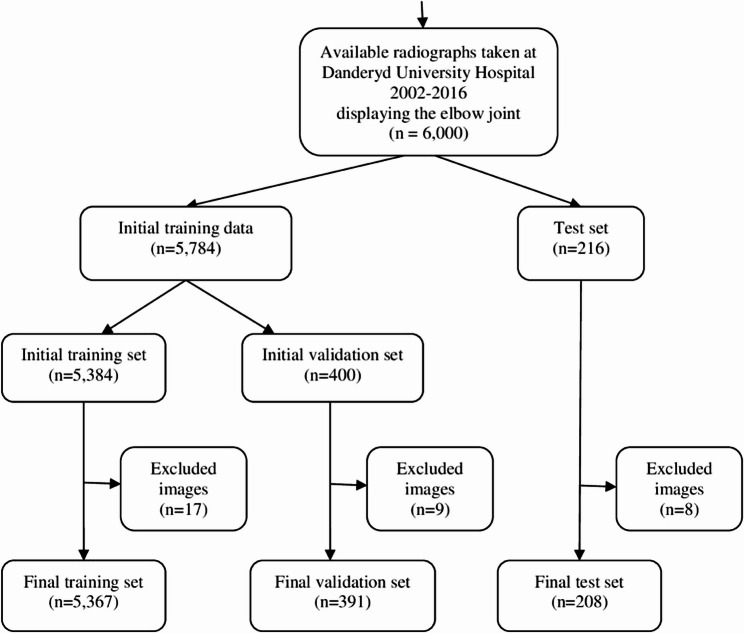
Flowchart of data sets. This chart shows dataset selection, number of cases initially available, and number of cases selected for each set. The number of excluded cases due to open physes or poor image quality is also shown. The arrows demonstrate unidirectional flow of radiological images throughout the course of the study

From 18,453 available elbow radiograph examinations 5,367 were used in the training set, 208 for the test set, and 391 were used for the validation set (Fig. 1).

In total 17 images during training, 9 images from validation, and 8 images from the test exams were excluded during classification. This is mainly because they showed elbows with open physes (which are classified differently) or because the image quality was too poor for fracture classification. Network performance was evaluated for a total of 54 fracture categories. The general fracture detection ability for all fractures around the elbow was 0.88 (95% CI 0.83–0.93), but this included diaphyseal fractures since these are often seen in radiographs displaying the elbow joint, which are not included in this study since they do not directly affect the elbow joint.

### Proximal radius (AO/OTA 2R1)

In total 798 (65.4%) training cases and 28 (43.8%) evaluation cases were considered as proximal radius fractures, with most fractures belonging to A2 and B1 classes. The overall category match was 0.89 (95% CI 0.81–0.98) with a frequency-weighted average AUC of 0.80. The AUC for the B group, mason fractures, was 0.89 (95% CI 0.77-1.00) while A (neck fractures) reached only an acceptable level with AUC 0.70 (95% CI 0.46–0.93) (Table 1), despite having a similar amount of training cases.

**Table 1:** Network performance for proximal radius. This table shows network performance for the different AO/OTA classes for proximal radius fractures, as well as customized fracture descriptors. Letter corresponds to fracture type and number to group. Dislocated is a customized class. AUC = Under the curve, ci = confidence interval

Proximal radius					
	Observed cases (*n* = 208)	Sensitivity (%)	Specificity (%)	Youden J	AUC (95% CI)
A	8	62	86	0.48	0.70 (0.46–0.93)
2	7	71	88	0.59	0.73 (0.46–1.00)
B	12	83	95	0.79	0.89 (0.77–1.00)
1	9	89	88	0.77	0.88 (0.73–1.00)
3	3	100	64	0.64	0.75 (0.62–0.88)
C	8	100	46	0.46	0.75 (0.59–0.91)
1	3	100	68	0.68	0.85 (0.67–1.00)
3	5	60	80	0.40	0.67 (0.40–0.93)
Dislocated	11	91	79	0.70	0.85 (0.77–0.94)
Angulated	3	67	77	0.44	0.70 (0.37–1.00)

**Fig. 2:**
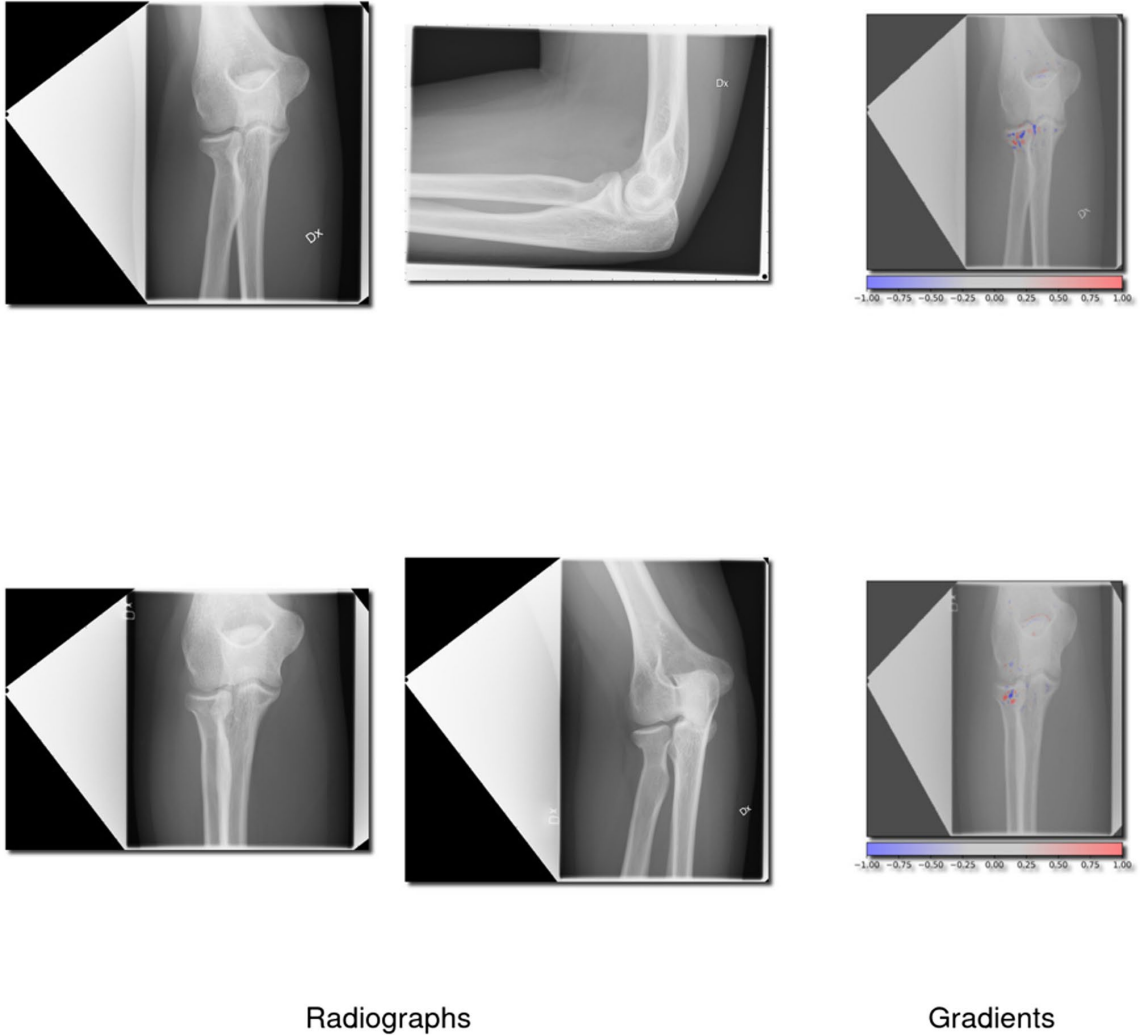
Correctly identified proximal radius (2R1A) fractures. This figure showcases 4 radiographs which the network correctly identified as a 2R1A fracture and 2 radiographs with gradients overlayed showing the network’s interpretation

**Fig. 3:**
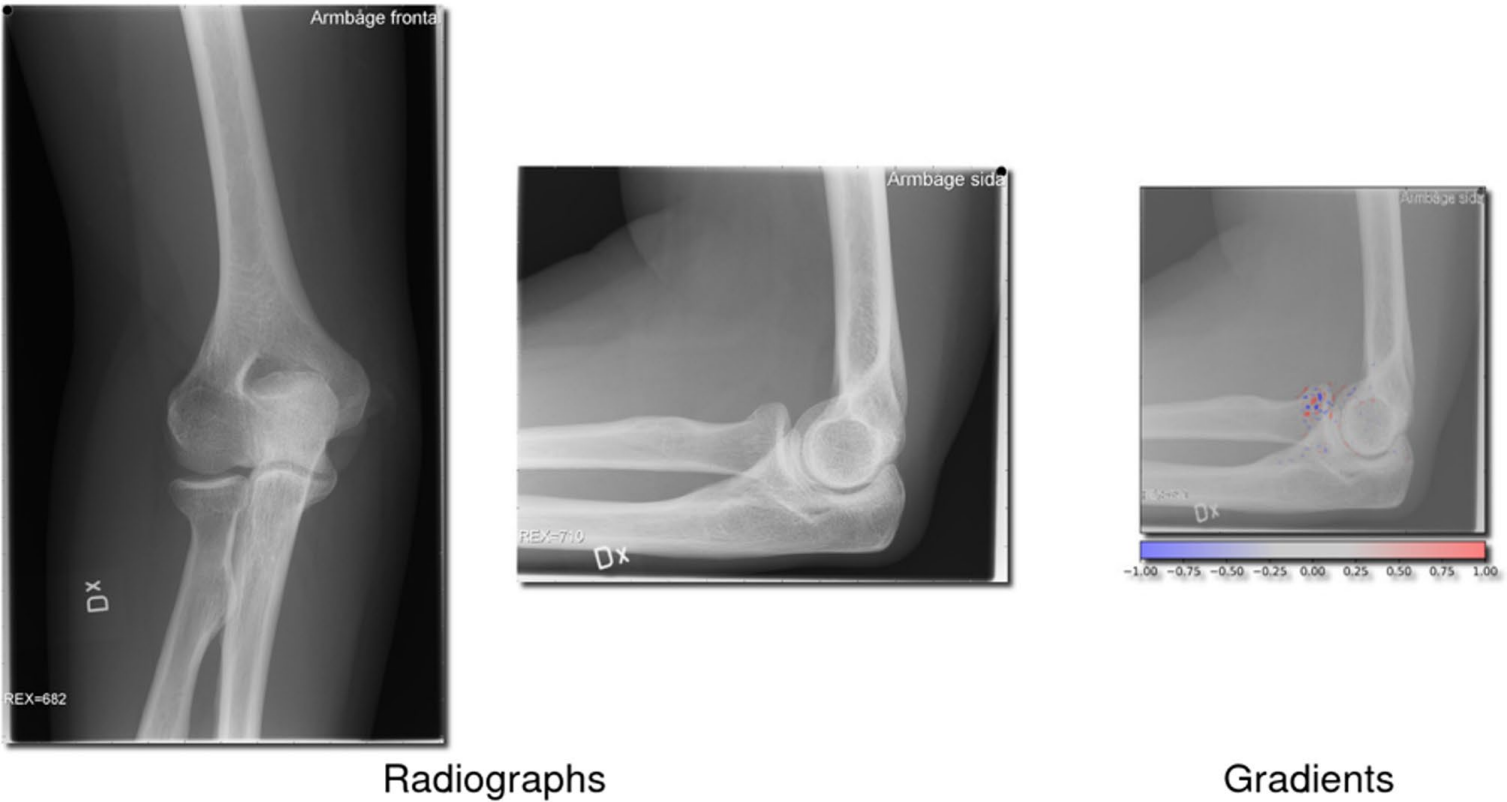
Missed proximal radius (2R1A) fractures. This figure showcases 2 radiographs in which the network failed to detect a 2R1A fracture, and 1 radiograph with gradients being overlayed showing the network’s interpretation

The C-group performed on par with the A-group but had only 12% of the training cases compared to the B-category. Examples of true positive cases (correctly identified fractures) and false negatives (missed fractures) are illustrated in Figs. 2 and 3, respectively.

### Proximal ulna (AO/OTA 2U1)

In total 510 (70.7%) training cases and 67 (79.8%) evaluation cases were assessed for proximal ulna fractures. The overall category match was 0.98 (95% CI 0.95-1.00) with a frequency-weighted average AUC of 0.86. The system performed outstandingly on type B1 fractures, commonly known as olecranon fractures, with an AUC of 0.92 (95% CI 0.87–0.97). Conversely, coronoid fractures, type B2, performed only acceptably with a mean AUC of 0.77 (95% CI 0.60–0.93) (Table 2).

**Table 2:** Network performance for proximal ulna. This table shows network performance for the different AO/OTA classes for proximal ulna fractures, as well as customized fracture descriptors. The first letter corresponds to fracture type, number to subgroup, and the last letter to the qualifier. Dislocated is a customized class. AUC = Under the curve, ci = confidence interval

Proximal ulna					
	**Observed cases** (*n* = 208)	**Sensitivity (%)**	**Specificity (%)**	**Youden J**	**AUC (95% CI)**
A	6	50	79	0.29	0.57 (0.30–0.85)
1	2	100	36	0.36	0.58 (0.13–1.00)
B	56	89	74	8.63	0.88 (0.83–0.94)
1	43	88	85	0.74	0.92 (0.87–0.97)
…d (simple)	21	86	66	0.51	0.77 (0.68–0.86)
…e (multifragmentary)	22	91	88	0.79	0.94 (0.89–0.99)
2	13	69	88	0.57	0.77 (0.60–0.93)
… n (sublime facet)	3	67	83	0.50	0.62 (0.11–1.00)
… o (tip)	4	50	94	0.44	0.63 (0.23–1.00)
… p (< 50%)	4	100	72	0.72	0.85 (0.71–0.99)
Dislocated	45	73	91	0.65	0.89 (0.83–0.94)

**Fig. 4:**
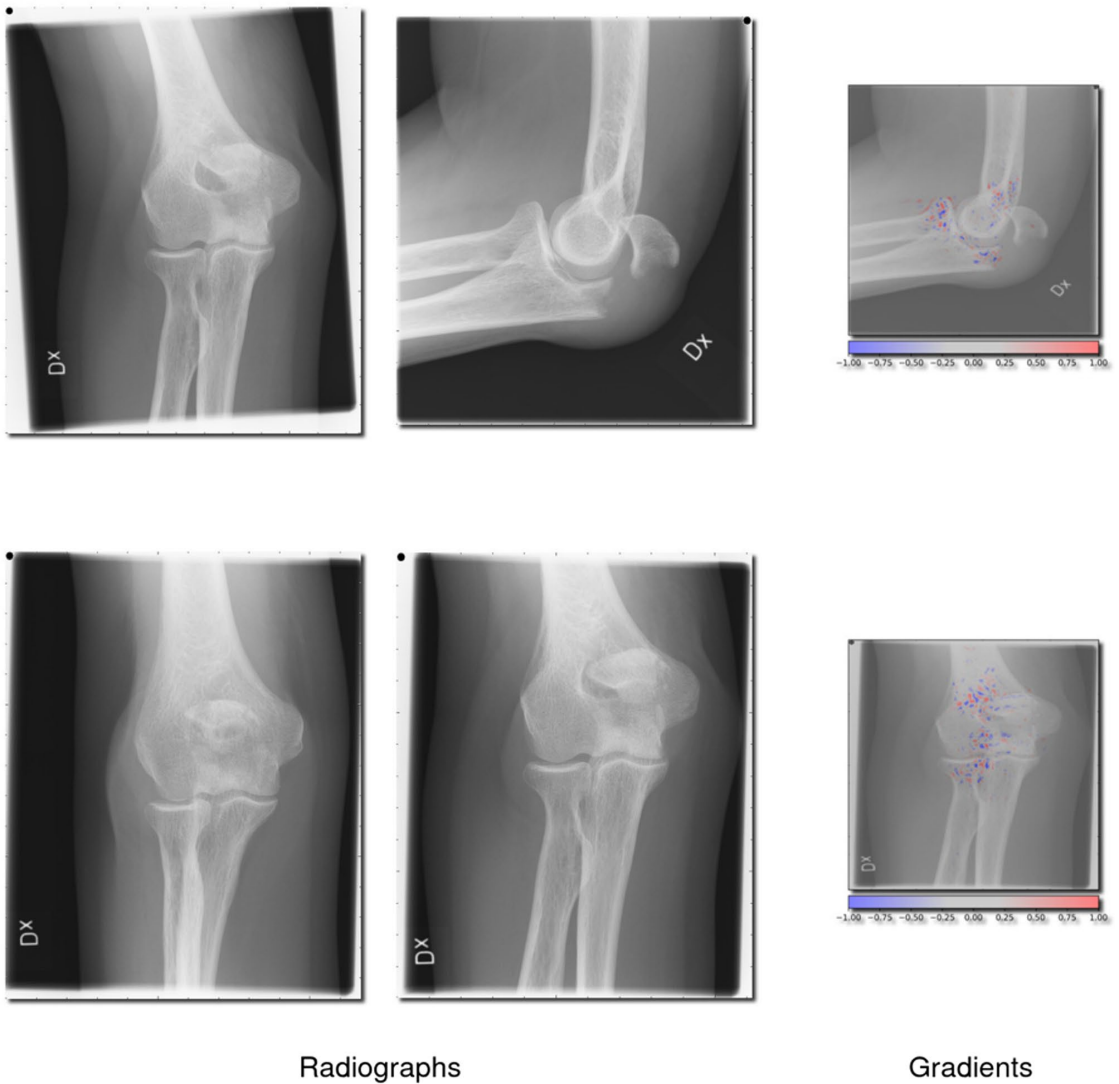
Correctly classified olecranon fractures (2U1B1). This figure showcases 4 radiographs where the network correctly identified a 2U1B1 fracture as being present, as well as 2 radiographs with gradients overlayed showing the network’s interpretation

**Fig. 5:**
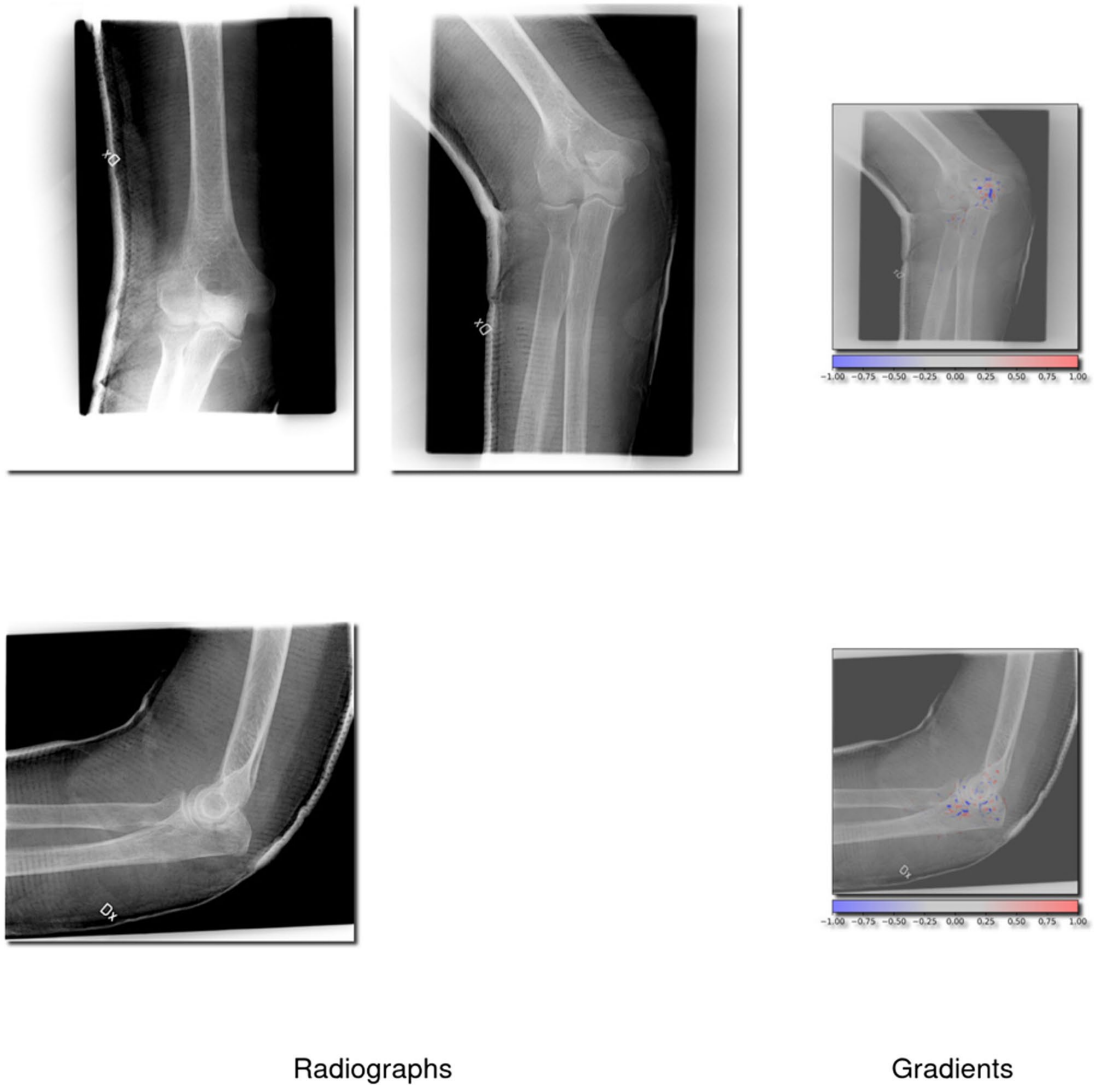
Missed olecranon fractures (2U1B1). This figure showcases 3 radiographs in which the network failed to identify a 2U1B1 as being present, also 2 radiographs with gradients overlayed showing the network’s interpretation

Examples of cases where B1 fractures were correctly identified can be seen in Fig. 4, and cases where they were missed can be seen in Fig. 5.

### Distal humerus (AO/OTA 13) 

The distal humerus fractures were the smallest group with only 286 (86.4%) training cases and 30 (90.9%) evaluation cases. The overall category match was 0.96 (95% CI 0.86-1.00) with a frequency-weighted average AUC of 0.85. Complete articular type C fractures performed the best with 0.92 (95% CI 0.84-1.00) although some certain subgroups in A and B also reached high AUC estimates (Table 3). 

**Table 3:** Network performance for distal humerus. This table shows network performance for the different AO/OTA classes for distal humerus fractures. Letter corresponds to fracture type, the first number to group, and the second number to subgroup. AUC = Under the curve, ci = confidence interval

Distal humerus					
	Observed cases (*n* = 208)	Sensitivity (%)	Specificity (%)	Youden J	AUC (95% CI)
A	15	80	75	0.55	0.82 (0.72–0.91)
1	8	88	67	0.54	0.77 (0.65–0.90)
…1	4	100	83	0.83	0.90 (0.83–0.97)
…2	4	75	68	0.43	0.71 (0.49–0.94)
2	4	100	78	0.78	0.91 (0.82–1.00)
…3	3	100	79	0.79	0.90 (0.79–1.00)
3	3	100	96	0.96	0.98 (0.95–1.00)
B	5	100	50	0.50	0.78 (0.59–0.98)
1	2	100	64	0.64	0.67 (0.58–0.76)
3	2	100	90	0.90	0.90 (0.86–0.94)
C	10	80	94	0.74	0.92 (0.84–1.00)
1	5	100	65	0.65	0.82 (0.68–0.96)
…1	3	100	53	0.53	0.74 (0.50–0.98)
2	4	100	91	0.91	0.97 (0.92–1.00)
…1	2	100	98	0.98	0.99 (0.97–1.00)
3	2	100	97	0.97	0.98 (0.95–1.00)

### Customized classes

Several customized classes were included in this study i.e. dislocation, effusion, arthritis, type of implant and old fracture. In total 15 custom classes that included 2 or more individual cases were assessed. The network overall performed accurately on these custom classes with an AUC above 0.9 for effusion, dislocation, and implants (Table 4).

**Table 4:** Network performance on customized classes. This table shows network performance for customized fracture qualifiers as well as other customized classes. AUC = Under the curve, ci = confidence intervals

Customized classes					
	**Observed cases** (*n* = 393)	**Sensitivity (%)**	**Specificity (%)**	**Youden J**	**AUC (95% CI)**
Dislocation	16	88	89	0.76	0.92 (0.84–1.00)
Effusion	47	100	91	0.91	0.96 (0.90–1.00)
Old fracture	13	46	96	0.43	0.68 (0.48–0.88)
Arthritis	19	84	77	0.61	0.86 (0.75–0.97)
- Inflammatory	4	75	73	0.48	0.68 (0.39–0.98)
- Osteoarthritis	13	85	67	0.51	0.73 (0.49–0.97)
… mild	9	89	90	0.79	0.92 (0.80–1.00)
… medium	5	100	64	0.64	0.80 (0.59–1.00)
… severe	5	100	64	0.64	0.79 (0.55–1.00)
Implant	13	85	99	0.84	0.94 (0.86–1.00)
- IM nail	2	100	91	0.91	0.91 (0.73–1.00)
- Plate	7	100	100	1.00	1.00 (1.00–1.00)
- Zuggertung	2	100	100	1.00	1.00 (1.00–1.00)

### Network insight and example images

Cases where the network showed the highest certainty for a prediction, regardless of if it was correct or not, were sampled for analysis. To understand how the network interprets these fractures, we have provided images of correctly and incorrectly identified proximal radius (2R1A) fractures (Figs. 2 and 3). We have also provided cases where olecranon fractures (2U1B1) were correctly and incorrectly identified (Figs. 4 and 5).

## Discussion

We found that deep learning can be taught to use the AO/OTA classification system to diagnose elbow fractures in adults with an accuracy ranging from acceptable to excellent for most fracture classes. Subtle fractures such as coronoid (2U1B2) fractures were expectedly more challenging for the network. Custom classes such as “old fracture” also had a tendency towards somewhat lower AUC values, presumably due to relatively low amounts of cases. Old fractures could also be more difficult to discern given their presentation on radiography.

To our knowledge, the only previous attempt at using artificial intelligence for the detection of elbow fractures was in children [10]. Our approach to using multiple classes both for diagnosing and guiding treatment for elbow fractures is the first of its kind. Previous studies have been made in a similar way for the classification of ankle and knee fractures with very good results [12, 17]. With 54 fracture categories, where more than half of AUC estimates were above 0.8, we see these results as quite auspicious. While our study demonstrates promising outcomes in utilizing deep learning neural networks for diagnosing elbow fractures, a more cautious interpretation is advisable. Further research, including external validation and extensive clinical assessments, is needed to establish the full reliability and applicability of our approach in diverse settings. Nonetheless, we do believe in the use of AI becoming more mainstream in this field and used more the further this technology develops in the near future.

Studies using deep learning for fracture diagnostics have reported a higher overall diagnostic accuracy than our study. In the systematic review by Langerhuizen et al 2019 [11], they found six studies using neural networks to identify fractures on plain radiographs which reported AUC ranging from 0.95-1.0 [6, 7, 9, 21–23]. The difference in AUC could, as previously suggested by Lind et al. [17], be due to the high level of complexity of the joint and hence a much higher number of nested fracture categories. It should also be noted that our study includes 54 fracture categories and 15 customized classes, while Chung et al. [9] only had a total of 4 categories. We included pictures with distracting elements, such as implants and casts, which were excluded from the study by Urakawa et al [22]. This could be one explanation as to why cases such as the ones provided in Figure 5 were not correctly classified. In comparison to studies made using the same deep neural network and setup as this study, the results are quite similar with a majority of AUC estimates over 0.8 or 0.9 [12, 17]. For future comparison, our test set will be made public on the AIDA Data Hub.

To capture as many AO/OTA classes as possible, we actively selected rare fracture patterns since we believe that these rare fracture patterns are the ones where clinicians are most uncertain and in the greatest need of computer-assisted interpretation. This will hopefully decrease the risk of fracture patterns such as the “terrible triad” to be missed and thus, over time, the risk of serious complications. This could however be considered a limitation, as suggested in the article by Lind et al. [17] since a bias towards uncommon fracture patterns is introduced. It should also be noted that classifying discrete changes and unusual classes into their respective categories was a difficult task possibly allowing human error to affect the results. We also found that most of the poorly performing classes in this study were non-displaced or small and did not require specific treatment. However, what we believe is of utmost importance, is selecting the appropriate treatment method for certain fracture types, such as olecranon fractures. For these types of fractures, the model performed better, further proving the accuracy with which AI can improve patient outcomes.

We did not compare the neural network's performance to clinicians in this study. In a similar study on proximal humerus fracture classification, Chung et al found that orthopedics subspecialized in shoulder surgery performed with an AUC of 0.90-0.98 [9]. While we cannot directly compare our studies due to differences in the specific fracture locations and the number of classes used, we can offer some possible explanations for the difference in AUC values. The AUC for proximal radius B-fractures was 0.89, and the Kappa was 0.72. However, or the B3 subgroup, while the Kappa was high, the AUC was lower (0.75), likely due to fewer training examples and wide confidence intervals. This suggests alignment between human and model uncertainty. To confirm this observation, it is important that future studies include comparison experiments with orthopedic surgeons or radiologists. In addition, integrated gradients are limited in their interpretability and may be prone to misleading attention to non-fracture features (e.g., laterality markers). In this study, we primarily used gradients to verify that the model focused on the correct anatomical region. We believe clinical trust is built more on familiarity with the classification system and the ability to visually assess the suggested class. This could have practical clinical benefit of AI assistance is narrowing down possible fracture types to a short, plausible list.

Furthermore, it is important to note that in our data division, patients may appear multiple times in the same set, such as the test set, due to the random division method. While this design choice allows for the inclusion of patients on multiple occasions, it is without overlap between the test, training, and validation sets. The extent to which patients are represented multiple times in the test set was not specifically quantified in this study and may vary. Patients were split such that no individual appeared in both training and validation sets. Since patients could only be included once every 90 days, repeated instances should be rare. While it is possible that certain patients are prone to similar injuries, our clinical experience is that bilateral elbow fractures of the same type are uncommon. Nevertheless, this aspect should be considered when interpreting the generalizability of our findings and is acknowledged as a potential limitation in the methodology employed.

One possible explanation is that elbow fractures may inherently be more complex and difficult to diagnose than proximal humerus fractures, at least when considering the number of AO/OTA classes. Additionally, our study included rare and complex fracture patterns, while Chung et al.'s study focused on a more common fracture pattern. These rare fracture patterns were deliberately selected in order to capture the most difficult and uncertain cases, making our results more representative of the complexity and variety of fractures that clinicians encounter in practice, which may have contributed to the lower AUC values.

Despite the difference in AUC values, we believe that our study's results still demonstrate the potential for AI to aid clinicians in the interpretation and classification of fractures around the elbow joint. Additionally, the sample size for rare fractures was small and future implementation studies should prioritize an expanded sample size in order to improve performance in classifying rare fractures.

Another limitation of this study might be that it was performed at a single site. This may limit the potential for this specific network to be applied at another hospital with the expectation to achieve similar diagnostic performance. We recommend that future studies include external validation in order to improve the clinical usability of the network. Ideally, geographical external validation should be used to assess performance and generalizability [24].

 This study provides a diagnostic tool that identifies if a fracture is present or not and generates a detailed classification for that fracture. This is information that might not normally be a radiologist’s area of expertise but is very useful for an orthopedic surgeon in deciding how to treat the patient. This should prove useful during night shifts when, as previously discussed, the risk of mistakes is higher [25].

In conclusion, we found artificial intelligence through deep learning can be taught to use the AO/OTA classification system to diagnose elbow fractures in adults with an accuracy ranging from acceptable to excellent for most fracture classes. 

## Supplementary Information

Supplementary Material 1.

## Data Availability

Data is available from the corresponding author on reasonable request.
