# Supplementary figures and images for: Use of artificial intelligence for classification of fractures around the elbow in adults according to the 2018 AO/OTA classification system
Source: BMC Musculoskelet Disord. 2025 Sep 9;26:848. doi: 10.1186/s12891-025-09161-2 (PMC12418695; doi:10.1186/s12891-025-09161-2)

Supplemental figure 1: Logical diagram of the algorithm used in the study.


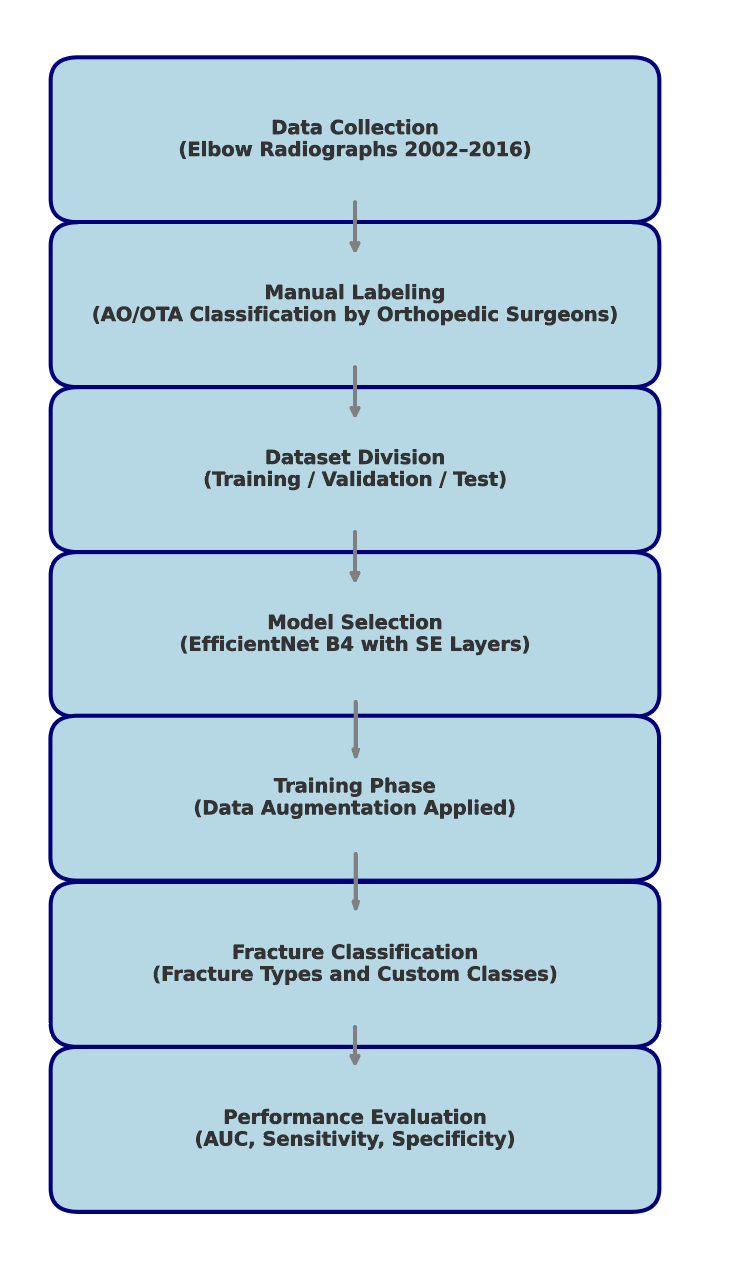

Supplement: Supplementary file 1 — Supplementary Material 1. [file 12891_2025_9161_MOESM1_ESM.docx]
